# A novel stop-loss variant in ISG15 causes C-terminal dysfunction and impairs USP18-mediated negative feedback

**DOI:** 10.3389/fimmu.2026.1916670

**Published:** 2026-09-17

**Authors:** Wanyu Zhang, Xiongbin Chen, Yulu Li, Xingfeng Yao, Jinyan Huang, Yijiu Gao, Wenlu Xing, Dan Lu, Mengyue Deng, Zhou Shu, Huawei Mao

**Affiliations:** 1 Department of Rheumatology and Immunology, Beijing Children’s Hospital of Capital Medical University, National Center for Children’s Health of China, Beijing, China; 2 Department of Pathology, Beijing Children’s Hospital, Capital Medical University, National Center for Children’s Health (NCCH), Beijing, China; 3 Ministry of Education Key Laboratory of Major Diseases in Children, Beijing, China; 4 Beijing Key Laboratory for Genetics of Birth Defects, Beijing, China; 5 Beijing Key Laboratory of Precision Diagnosis and Treatment of Pediatric Immune Diseases, Beijing, China

**Keywords:** baricitinib, ISG15, stop-loss variant, type I interferonopathy, USP18

## Abstract

**Introduction:**

ISG15 is a type I interferon (IFN-I)-inducible ubiquitin-like protein that restrains IFN-I signaling. Classical ISG15 deficiency is thought to cause disease mainly by disrupting USP18 stabilization, which leads to sustained IFN-I signaling and interferon-driven autoinflammation. Whether patient-derived C-terminal ISG15 variants can impair IFN-I negative feedback through altered C-terminal functional presentation has remained unclear.

**Methods:**

We studied a Chinese boy with early-onset recurrent fever, severe ulcerative skin lesions, and elevated liver enzymes. Whole-exome sequencing was performed to identify ISG15 variants. Patient peripheral blood mononuclear cells (PBMCs) and skin tissue were analyzed for IFN-I pathway activation. Functional effects of the identified variants were assessed in 293T cells, and structural analysis was performed. The patient was treated with the JAK inhibitor baricitinib.

**Results:**

Two novel compound heterozygous ISG15 variants were identified: c.234C>A, which produced a truncated ISG15 protein, and c.498A>T, a stop-loss variant predicted to extend the ISG15 C-terminus and associated with markedly impaired ISGylation. Patient PBMCs and skin tissue showed increased p-STAT1 signaling and IFN-I pathway activation, with decreased USP18 expression and altered ISG15-USP18 regulation in PBMCs. In 293T cells, c.498A>T alone did not reduce USP18 abundance, suggesting that this variant contributes to disease through impaired C-terminal functional presentation rather than USP18 loss alone. Structural analysis suggested that the C-terminal extension may alter LRLRGG motif presentation, weakening ISG15-USP18-mediated negative feedback. Baricitinib reduced IFN-I-related markers and was associated with improvement of ulcerative skin lesions and liver enzyme abnormalities.

**Discussion:**

This study expands the genetic and mechanistic spectrum of ISG15-associated type I interferonopathy, and supports that JAK-STAT inhibition with baricitinib can effectively control IFN-I-driven inflammation.

## Introduction

Type I interferons (IFN-I) are central to antiviral defense and innate immune homeostasis. Upon binding to the interferon-α/β receptor (IFNAR), IFN-I activates the JAK1/TYK2-STAT1/STAT2-IRF9 cascade and induces a broad set of interferon-stimulated genes (ISGs) (1). Sustained activation of this pathway can promote chronic inflammation and tissue injury, so IFN-I signaling is subject to tight regulation. Type I interferonopathies are a group of monogenic disorders characterized by excessive IFN-I production or abnormal cellular responses to IFN-I. These diseases present with marked clinical heterogeneity, often affecting the skin, central nervous system and lungs (2, 3). Because clinical manifestations vary widely, early genetic diagnosis can help establish the molecular cause and guide targeted treatment (4).

Type I interferonopathies can arise from defects in nucleic acid metabolism or sensing, increased signaling downstream of IFNAR, or impaired negative feedback regulation (5). In the IFN-I pathway, USP18 serves as a STAT2-dependent negative regulator of the IFNAR complex, limiting prolonged JAK-STAT activation and excessive ISG expression. Pathogenic variants in USP18, ISG15 or specific regions of STAT2 can disrupt this negative feedback loop, leading to persistent IFN-I signaling (6, 7). *ISG15* encodes an IFN-I-inducible ubiquitin-like protein with several immune functions. As a secreted molecule, it contributes to IFN-γ-dependent anti-mycobacterial immunity. In its free intracellular form, it stabilizes USP18 and supports negative regulation of IFN-I signaling. In addition, ISGylation modifies target proteins involved in intracellular antimicrobial responses (8). These functions may explain why biallelic ISG15 deficiency can cause both susceptibility to mycobacterial infection and IFN-I-driven autoinflammation (8, 9). Clinical manifestations include intracranial calcification, pulmonary inflammation, and necrotic or ulcerative skin lesions (10–12).

Most reported cases of ISG15 deficiency are caused by truncating or other severe loss-of-function variants (13, 14). These variants are generally thought to impair IFN-I negative feedback by reducing USP18 abundance, as defective ISG15 can no longer maintain USP18 stability, resulting in sustained pathway activation. Whether an abnormal ISG15 C-terminus can compromise this feedback through altered LRLRGG presentation, in addition to or independent of USP18 abundance, has remained unclear in patients.

Here, we report a Chinese child with compound heterozygous *ISG15* variants, comprising a truncating variant c.234C>A and a stop-loss variant c.498A>T. The patient presented with early-onset, recurrent severe ulcerative skin lesions and systemic inflammation. By combining clinical phenotyping, IFN-I signaling assays, transcriptomic profiling, protein functional studies, and structural bioinformatics, we found that c.498A>T leads to a C-terminally extended ISG15 protein, impaired ISGylation and persistent IFN-I pathway activation. Patient PBMCs showed reduced USP18 expression, whereas c.498A>T alone did not decrease USP18 abundance in a 293T overexpression system, suggesting that this stop-loss variant may act through altered C-terminal function presentation rather than USP18 protein loss alone. We also assessed molecular and clinical responses before and after baricitinib treatment, which further supports targeting the JAK-STAT pathway in selected patients.

## Materials and methods

### Whole-exome sequencing and Sanger sequencing

Genomic DNA was extracted from peripheral blood samples from the proband and his parents. Whole-exome sequencing was performed by Kangso Medical Inspection (Beijing, China) using paired-end 150-bp sequencing on an Illumina platform, with an average sequencing depth of approximately 100×. Raw reads were aligned to the human reference genome GRCh37/hg19. Variants were annotated using ANNOVAR and filtered against public population databases, including gnomAD, the 1000 Genomes Project, ExAC and ESP6500. Candidate variants were validated by Sanger sequencing and familial co-segregation analysis. The stop-loss variant c.498A>T was further assessed using TAILVAR (v1.1), a machine-learning framework specifically developed for stop-loss variants with C-terminal extensions (15). Family history was obtained from the parents, including information on siblings and grandparents.

### Cell culture, plasmids and transfection

Human ISG15 WT, ISG15 c.234C>A, ISG15 c.498A>T and empty vector (EV) constructs were cloned into the pLVX-3×Flag-C vector. Co-transfection of the c.234C>A and c.498A>T constructs was used to model the compound heterozygous state. Additional plasmids included pcDNA3.1-His-UBA7, pcDNA3.1-3×Flag-UBE2L6, pcDNA3.1-Myc-HERC5 and pcDNA3.1-Myc-USP18. 293T cells were cultured in DMEM supplemented with 10% FBS and transfected using Hieff Trans Universal Transfection Reagent (Yeasen, China).

### PBMCs isolation and IFN-α stimulation

PBMCs were isolated from fresh heparin-anticoagulated whole blood by density gradient centrifugation. Cells were resuspended in RPMI 1640 medium supplemented with 10% FBS and stimulated with 100 ng/mL recombinant human IFN-α(AbMole, M20003) for 0, 15, 60 or 120 min, 12 h, 24 h, or 48 h before collection.

### JAK inhibitor treatment *in vitro*

PBMCs were stimulated with CD3/CD28 beads and cultured with 200 IU/mL IL-2 to generate activated T cells. Activated T cells were then transferred to IL-2-free complete medium and pretreated with 0.1 or 1 μM baricitinib (MCE, HY-15315) or DMSO for 4 h, followed by stimulation with or without 100 ng/mL IFN-α for 15 min. 293T cells were treated with 1 μM baricitinib or DMSO for 4 h at 48 h after transfection and then harvested.

### Flow cytometric analysis of phospho-STAT1

PBMCs were first stained with CD14-FITC for surface marker detection. PBMCs or activated T cells were then stimulated with IFN-α and fixed with BD Phosflow Fix Buffer I, followed by permeabilization with BD Phosflow Perm Buffer III. Cells were incubated with anti-phospho-STAT1 (Tyr701) antibody (CST, 9167S) or an isotype control, followed by Alexa Fluor 647-conjugated secondary antibody. Samples were acquired on a Beckman flow cytometer and analyzed using FlowJo.

### RNA sequencing and differential expression analysis

Total RNA was extracted from PBMCs using TRIzol. Library preparation and sequencing were performed by Tsingke Biotechnology (Beijing, China) using the VAHTS Universal V6 RNA-seq Kit on an Illumina NovaSeq X Plus platform with paired-end 150-bp reads. Each RNA sample underwent two independent library preparations. Raw reads were filtered with fastp and aligned to the GRCh38.112 reference genome using HISAT2. Differential expression analysis was performed with DESeq2 using raw read counts. Differentially expressed genes were defined by |log2FC| > 1 and Benjamini-Hochberg adjusted P < 0.05. GO and KEGG enrichment analyses were then performed.

### RT-qPCR and IFN-I score calculation

Total RNA was extracted using TRIzol and reverse-transcribed with Hifair V one-step RT-gDNA digestion SuperMix (Yeasen, China). qPCR was performed using Hieff UNICON Advanced qPCR SYBR Master Mix (Yeasen, China), with GAPDH as the internal control. Relative expression was calculated using the 2^−ΔΔCt^ method. Primer sequences are listed in **Supplementary Table 3**. The IFN-I score was calculated as previously described (16). Briefly, raw RNA-seq read counts were normalized using DESeq2 size factors. For each gene, a Z-score was calculated using the mean and standard deviation of healthy controls as the reference. The IFN-I score for each sample was defined as the sum of Z-scores across the 28-gene panel, with higher scores indicating stronger activation of the type I interferon signature.

### Histology and immunohistochemistry

Paraffin-embedded sections were deparaffinized, rehydrated, subjected to antigen retrieval and blocked. Sections were incubated overnight at 4 °C with anti-phospho-STAT1 (Tyr701) antibody (CST, 9167S), followed by HRP-conjugated secondary antibody and DAB visualization. Sections were counterstained with hematoxylin and imaged using a bright-field microscope.

### Structural bioinformatics and molecular dynamics simulations

The predicted extension product of the ISG15 c.498A>T variant was inferred from the reference transcript ENST00000379389. This variant converts the canonical stop codon into a tyrosine codon and is predicted to generate a 21-amino-acid extension. A stop-loss ISG15 sequence was generated based on this predicted extension. WT and c.498A>T ISG15-USP18 complex models were generated with AlphaFold 3. Model confidence was assessed using AlphaFold confidence metrics, including interface predicted TM-score (ipTM), predicted TM-score (pTM), and predicted local distance difference test (pLDDT). The WT ISG15-USP18 model showed an ipTM of 0.79, a pTM of 0.79, and a mean pLDDT of 90.00, whereas the c.498A>T ISG15-USP18 model showed an ipTM of 0.66, a pTM of 0.66, and a mean pLDDT of 81.83. Structural alignment, Cα displacement, local cavity volume, residue exposure and contact network analyses were performed to assess local structural changes. The predicted WT and c.498A>T ISG15-USP18 complex models were then subjected to 100-ns molecular dynamics simulations using GROMACS with the Amber99sb force field and TIP3P water model. RMSD and RMSF were analyzed to evaluate complex stability and dynamics. Because structural modeling and molecular dynamics simulations are predictive approaches, these results were interpreted as hypothesis-generating rather than definitive structural evidence.

### Analysis of the ISG15 LRLRGG sequence and USP18 reference contact region

To assess the potential effect of the c.498A>T extension on the local spatial relationship between ISG15 and USP18, representative conformations at 0, 50 and 100 ns were analyzed. The WT ISG15-USP18 complex was used as the reference. USP18 residues within 4 Å of the GG residues in the C-terminal LRLRGG motif of WT ISG15 were defined as the USP18 reference contact residue set. Minimum heavy-atom distances between this residue set and the terminal GG residues of WT ISG15, or the corresponding GG residues within the LRLRGG sequence of c.498A>T ISG15, were then calculated and compared.

### Immunoblotting and Co-IP

Cells were lysed using standard lysis buffer, and proteins were analyzed by immunoblotting. Primary antibodies are listed in **Supplementary Table 4**. Co-immunoprecipitation was performed using the LABLEAD DYKDDDDK-Magnetic Immunoprecipitation Kit (PFM025). 293T cells were lysed 48 h after transfection. A fraction of the lysate was kept as input, and the remaining lysate was incubated with DYKDDDDK magnetic beads at 4 °C for 1 h. After magnetic separation and washing, bound proteins were eluted with SDS sample buffer and analyzed by immunoblotting.

### Statistics

Data are presented as mean ± SEM. Normality was assessed using the Shapiro-Wilk test, and equality of variance was assessed using the Brown-Forsythe test. For normally distributed data, two-group comparisons were performed using an unpaired two-tailed Student’s t-test when variances were equal, or Welch’s t-test when variances were unequal. P < 0.05 was considered statistically significant.

## Results

### Clinical presentation and identification of compound heterozygous *ISG15* variants

We enrolled a Chinese boy who got sick very early in infancy. He presented with recurrent fever and inflammatory skin lesions that initially appeared as reticular and branching erythema, followed by subcutaneous induration, necrosis, ulceration, crusting and scarring. The lesions mainly involved the groin, lower abdomen and axillae (**Figure 1A**). He had no photosensitivity, arthritis or pulmonary involvement (**Table 1**). Head CT and brain MRI showed no intracranial calcification or obvious structural abnormality (**Figure 1A**; **Table 1**). He developed a normal scar after BCG vaccination, and tuberculosis screening was negative. Laboratory testing revealed systemic inflammation, intermittent elevation of liver enzymes and reduced complement levels, whereas autoantibodies were negative. Peripheral lymphocyte and helper T-cell subset analysis did not show severe immune cell deficiency, but revealed altered B-cell maturation/memory proportions and a reduced proportion of Th2-like cells (**Supplementary Table 1** and **S2**). Skin histopathology showed focal tissue necrosis, inflammatory cell infiltration and vasculitis/panniculitis-like changes (**Figure 1B**), supporting an early-onset autoinflammatory disorder with predominant cutaneous involvement.

**Figure 1:**
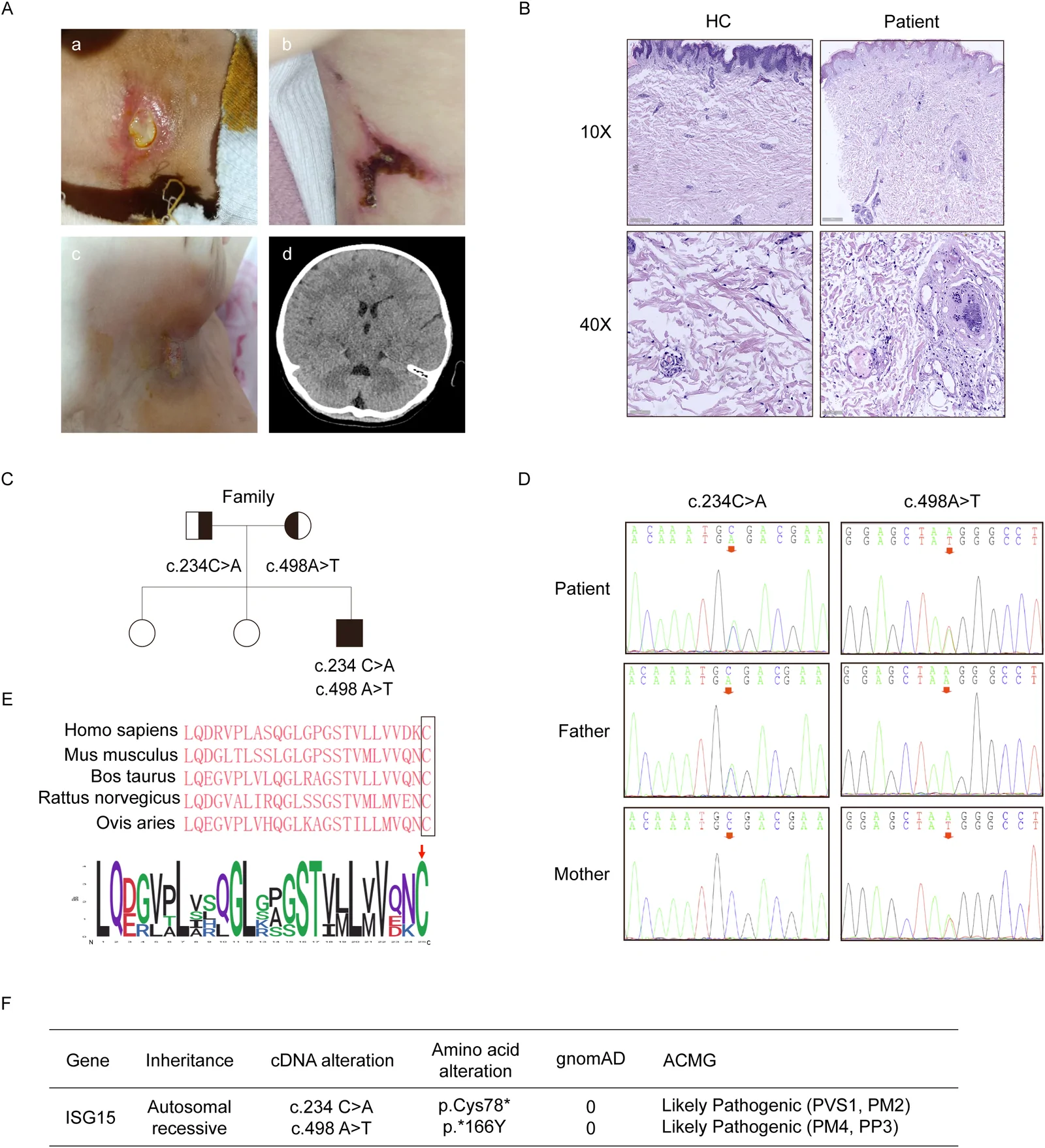
Type I interferonopathy in a patient with compound heterozygous ISG15 variants. **(A)** Pedigree of the family showing compound heterozygous ISG15 variants c.234C>A and c.498A>T in the patient. **(B)** Sanger sequencing validation of the ISG15 variants in the family. **(C)** Cross-species conservation of the ISG15 c.234C>A/ p.Cys78* site. **(D)** Clinical photographs and cranial CT of the patient. Extensive ulcerative skin lesions were present in the axillae, lower abdomen and groin (a-c); cranial CT showed no intracranial calcification (d). **(E)** H&E staining of patient skin tissue. Scale bars, 200 μm and 50 μm. **(F)** Population frequency and ACMG classification of the ISG15 variants.

**Table 1:** Clinical and laboratory features of the patient.

Demographics		
Sex	Male	
Age at first presentation	9 months	
Ethnicity	Chinese	
Clinical features		
Skin manifestations	Axillae/Groin/Abdomen	
Necrosis	Yes	
Ulceration	Yes	
Reticulated erythema	Yes	
Hyperpigmentation	Yes	
Scarring	Yes	
Photosensitivity	No	
Arthritis	No	
Recurrent fevers	Yes	
Neurological involvement	None	
Lung involvement	None	
Growth	-1 SD	
BCG vaccination reaction	Normal BCG scar	
T-SPOT/PPD	Negative	
Imaging		
Neuroimaging	CT-brain-normal MRI brain-normal	
Abdominal CT	Hepatomegaly and mild abdominal/retroperitoneal lymphadenopathy; otherwise normal solid organs.	
Chest CT	Unremarkable	
Blood tests	Pre-baricitinib	Post-baricitinib
White blood cell (4-10 10^9^/L)	10.99	7.18
Neutrophils (0.72-4.6 10^9^/L)	4.43	2.6
Lymphocytes (0.72-4.6 10^9^/L)	5.5	2.95
Red blood cell (3.5-5.5 10^12^/L)	4.34	3.98
Haemoglobin (110–160 g/L)	107	113
Platelets (100-400 10^9^/L)	364	251
CRP (<10 mg/L)	21.26	<10
PCT (0-0.5 ng/mL)	0.8	0.24
ESR (0–15 mm/h)	3	4
Liver function tests	Pre-baricitinib	Post-baricitinib
AST (5–40 U/L)	223.1	67.9
ALT (5–40 U/L)	232.3	43.5
Complement function	Pre-baricitinib	Post-baricitinib
C3 (0.89-1.87 g/L)	0.7	0.97
C4 (0.14-0.44 g/L)	0.12	0.255
Autoantibodies	Pre-baricitinib	Post-baricitinib
ANA (< 1:40)	Negative	Negative
Anti-dsDNA (0–10 IU/mL)	Negative	Negative
Anti-Sm (0–20 RU/mL)	Negative	Negative

Whole-exome sequencing identified compound heterozygous ISG15 variants, c.234C>A and c.498A>T. The c.234C>A variant introduced a premature stop codon at p.Cys78, whereas c.498A>T was a stop-loss variant predicted to extend the C-terminus of ISG15. Both parents were unaffected heterozygous carriers, consistent with autosomal recessive inheritance (**Figure 1C**). Sanger sequencing confirmed that the two variants were inherited from different parents (**Figure 1D**). The father carried c.234C>A and the mother carried c.498A>T, neither parent had recurrent fever, ulcerative skin lesions, mycobacterial infection, pulmonary involvement or neurological abnormalities. The patient had two healthy elder sisters, and the grandparents were also reported to be healthy, with no similar disease in the family. The p.Cys78 residue was highly conserved across species (**Figure 1E**). Neither variant had a clear record in population databases. The c.498A>T variant, corresponding to chr1:965191A>T in GRCh38, had a TAILVAR score of 0.91, exceeding the recommended pathogenicity threshold (≥0.70), supporting a deleterious effect. According to ACMG guidelines, the c.498A>T variant was classified as likely pathogenic, with evidence supporting PM4 for a stop-loss change altering protein length and PP3 for predicted deleterious effects. In addition, the c.234C>A variant was classified as likely pathogenic (**Figure 1F**).

### Activation of type I interferon signaling in the patient

The clinical phenotype suggested dysregulated type I interferon signaling. After IFN-α stimulation, patient PBMCs showed higher p-STAT1 levels than healthy control cells (**Figure 2A**). Flow cytometry confirmed increased p-STAT1 in both total PBMCs and CD14^+^ monocytes from the patient, under basal conditions and after IFN-α stimulation (**Figure 2B**). p-STAT1 staining was also stronger in the patient’s skin tissue than in control skin (**Figure 2C**). In 293T cells, expression of patient-derived ISG15 variants increased p-STAT1 compared with WT ISG15, whereas baricitinib reduced this signal (**Figure 2D**). These findings indicate enhanced JAK-STAT pathway activation.

**Figure 2:**
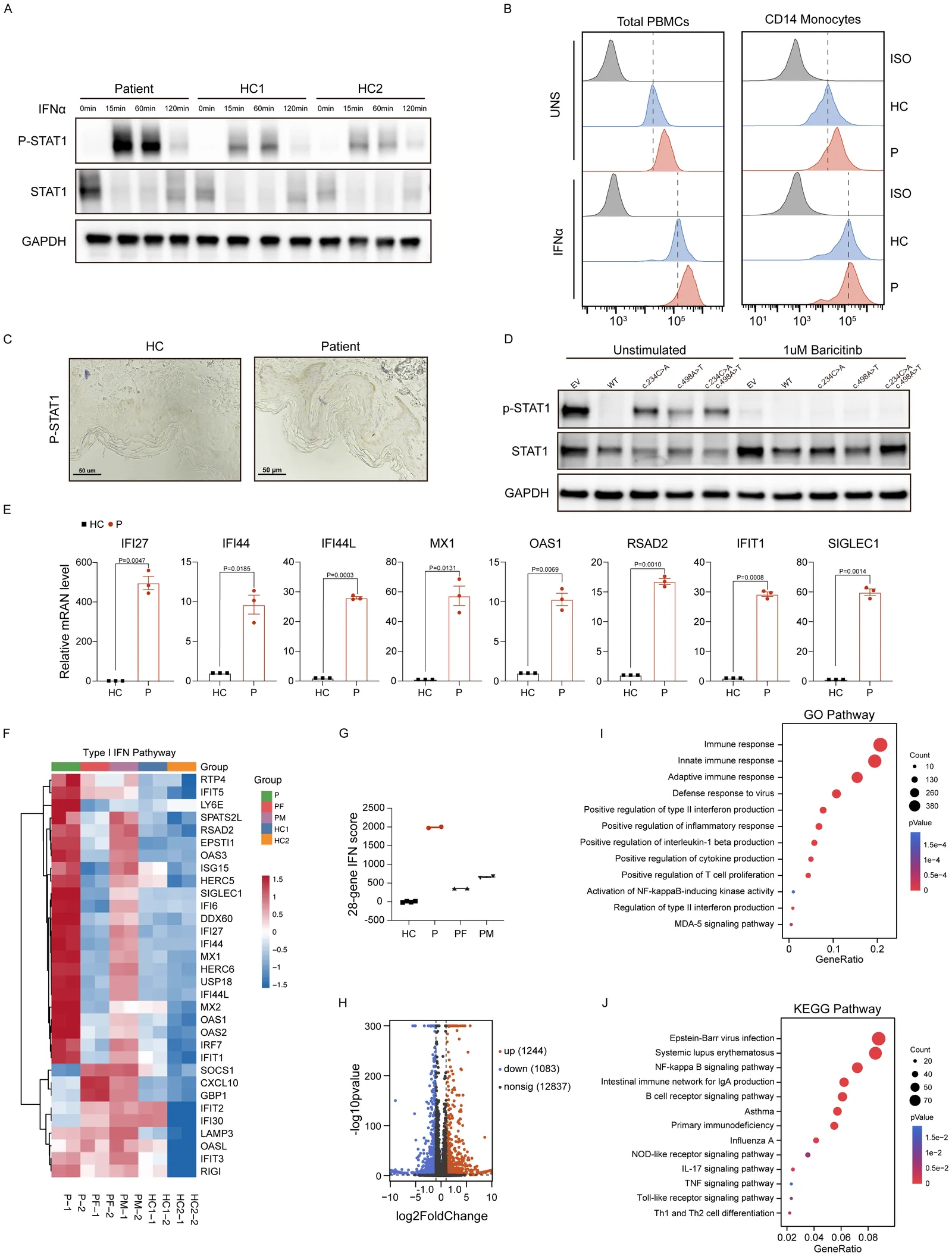
Hyperactivation of the type I interferon pathway in the patient. **(A)** Immunoblot analysis of p-STAT1 and STAT1 expression in PBMCs from the patient and healthy control (HC) after IFN-α stimulation. **(B)** Flow cytometric analysis of p-STAT1 levels in total PBMCs and CD14^+^ monocytes from the patient and HC. **(C)** Immunohistochemical staining of p-STAT1 in skin tissues from the patient and HC. Scale bar, 50 µm. **(D)** 293T cells were transfected with EV, WT, c.234C>A, c.498A>T or c.234C>A/c.498A>T, treated with baricitinib, and analyzed for p-STAT1 and STAT1 expression. **(E)** RT-qPCR analysis of ISG expression in PBMCs. **(F)** RNA-seq analysis showing the expression of IFN-I pathway-related genes in patients, their parents, and HC. **(G)** IFN-I scores in HC, the patient, and the patient’s parents. **(H)** Volcano plot of differentially expressed genes in the patient compared with HC. **(I, J)** GO and KEGG enrichment analyses. Data are presented as mean ± SEM. Statistical significance was determined using a two-tailed unpaired Welch’s t-test. Exact P values are indicated in the graphs.

We next assessed the downstream interferon signature. RT-qPCR showed increased expression of representative ISGs in patient PBMCs (**Figure 2E**). RNA-seq confirmed broad upregulation of IFN-I pathway-related genes, and the 28-gene IFN-I score was higher in the patient than in healthy controls (**Figures 2F, G**). We further incorporated RNA-seq data from patient’s parents, who were unaffected heterozygous carriers. The patient showed the strongest IFN-I signature, whereas both parents showed mild-to-moderate upregulation of a subset of IFN-I-related genes, with overall levels between healthy controls and the patient. GO and KEGG enrichment analyses identified immune response, antiviral response, NF-κB signaling, and cytokine-related pathways as enriched terms (**Figures 2H–J**). These results establish activation of the IFN-I pathway in the patient and provide transcriptomic comparison with parental heterozygous carriers.

### The ISG15 c.498A>T variant affect C-terminal LRLRGG presentation and local structure

ISG15 precursor protein is cleaved at its C-terminus to expose the LRLRGG sequence and generate mature ISG15 (**Figure 3A**). The c.234C>A variant terminates early, so UBL2 and the C-terminal LRLRGG sequence are lost. The c.498A>T variant is a stop-loss variant predicted to produce a C-terminal extension (**Figure 3A**). Structural analysis based on the predicted extension model revealed local changes around the LRLRGG region compared with WT ISG15. These included Cα displacement of residues 155-161, reduced local cavity volume, and altered residue contact patterns (**Figures 3B–E**). So the predicted C-terminal extension probably changes the local structure around the LRLRGG motif. ISG15 expression was reduced in patient PBMCs (**Figure 3F**). In 293T cells, both anti-Flag and the full-length anti-ISG15 antibody readily detected all three ISG15 variants, whereas the C-terminal antibody failed to recognize the truncated forms (**Supplementary Figures 1A–C**). The faint band observed in patient samples is therefore more likely due to low protein abundance than to antibody recognition failure.

**Figure 3:**
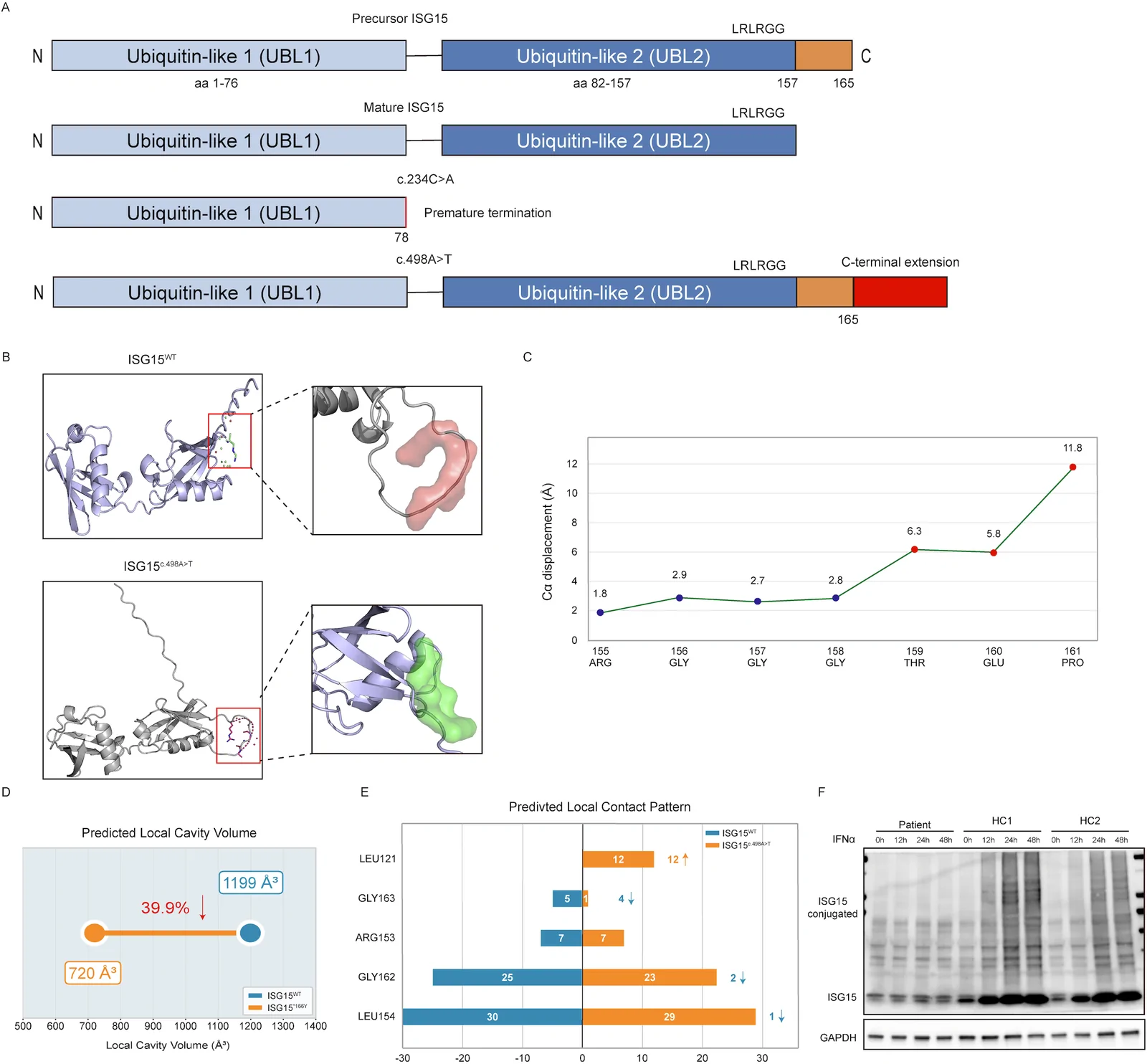
The ISG15 c.498A>T variant may affect C-terminal LRLRGG functional presentation and the structure of its adjacent region. **(A)** Schematic representation of precursor ISG15, mature ISG15 and the variants carried by the patient. **(B)** Structural models of WT and c.498A>T, with enlarged views of the LRLRGG-adjacent region. **(C)** Local Cα displacement of residues 155-161. **(D)** Comparison of local cavity volume. **(E)** Comparison of local residue contact patterns around the LRLRGG region. **(F)** Immunoblot analysis of ISG15 in PBMCs from HC and the patient after IFN-α stimulation.

### The ISG15 c.498A>T variant affect functional ISG15-USP18 regulation

Previous studies have shown that ISG15 supports USP18-mediated negative regulation through both maintenance of USP18 protein levels and non-covalent interactions involving the ISG15 C-terminal LRLRGG sequence. We therefore examined whether the c.498A>T variant could affect the ISG15-USP18 regulatory module. Predicted energy analysis indicated that the c.498A>T ISG15-USP18 model had lower interface energy and binding energy than the WT complex, suggesting that overall predicted binding was not weakened (**Figure 4A**). USP18 expression was decreased in patient PBMCs compared with healthy controls (**Figure 4B**). Co-IP performed in 293T cells overexpressing the corresponding plasmids showed that c.234C>A was associated with reduced USP18 protein levels; in contrast, c.498A>T alone neither reduced USP18 protein levels nor affected ISG15-USP18 binding (**Figure 4C**). These results distinguish the endogenous patient PBMC phenotype from the effect of the c.498A>T allele alone and suggest that reduced USP18 in the patient may be mainly related to the truncating c.234C>A allele.

**Figure 4:**
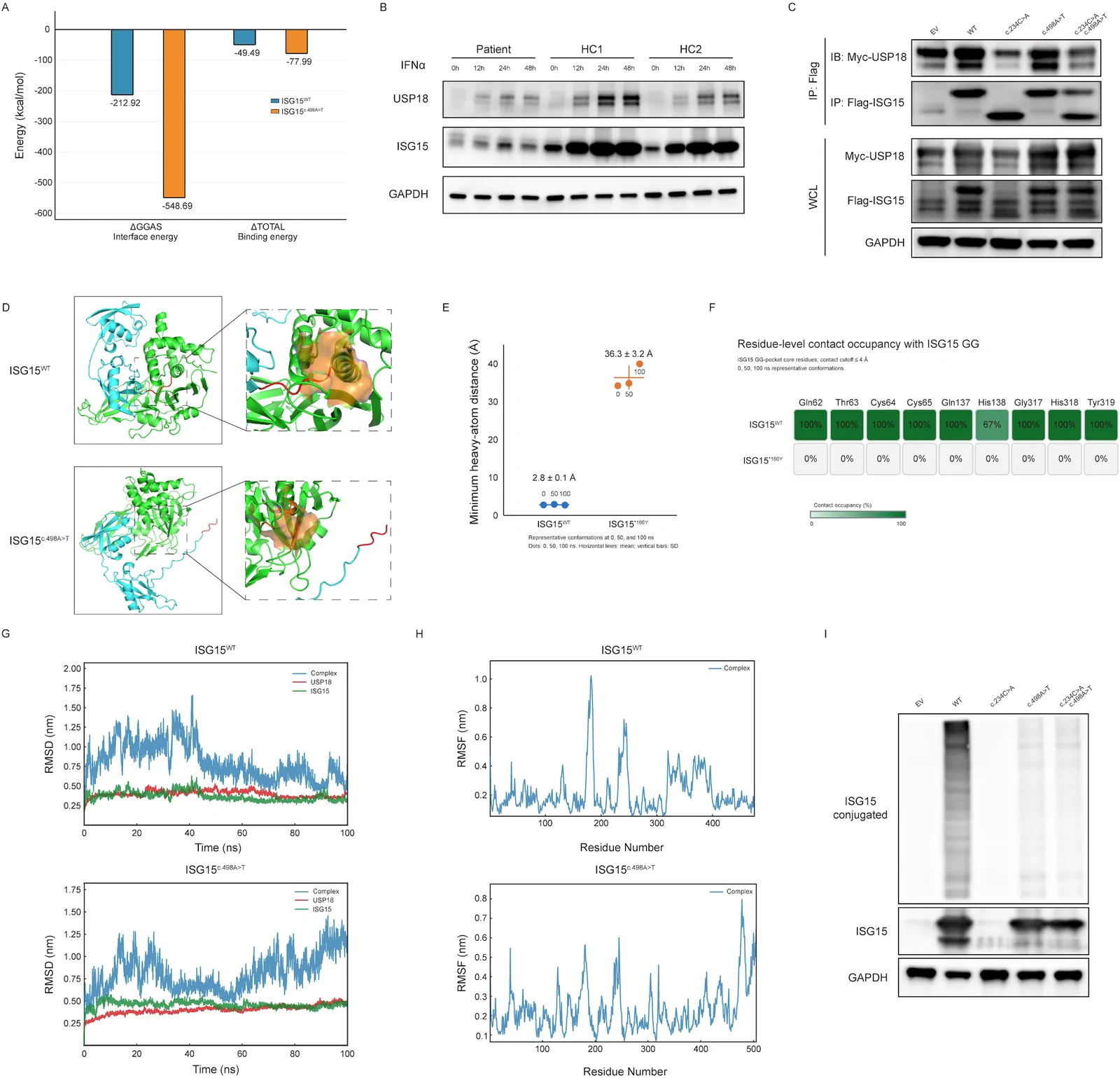
The ISG15 c.498A>T variant is associated with altered ISG15-USP18 regulation. **(A)** Predicted interface energy and binding energy of USP18 in complex with WT or c.498A>T. **(B)** Detection of USP18 and ISG15 in PBMCs from HC and the patient after IFN-α stimulation. **(C)** Co-IP analysis of the interaction between Myc-USP18 and Flag-tagged WT, c.234C>A, c.498A>T or c.234C>A/c.498A>T. **(D)** Structures of USP18 in complex with WT or c.498A>T. **(E)** Minimum heavy-atom distance between the terminal GG residues of WT ISG15 or the corresponding GG residues in the c.498A>T and the USP18 reference contact region. **(F)** Contact frequency between the GG residues and the USP18 reference contact region. **(G, H)** RMSD and RMSF analyses. **(I)** 293T cells were co-transfected with ISGylation system components and different ISG15 constructs, and ISG15-conjugated proteins and free ISG15 were detected by immunoblotting.

Structural modeling showed that c.498A>T shifted the LRLRGG region relative to the USP18 reference contact region (**Figure 4D**). In representative conformations at 0, 50 and 100 ns, the minimum heavy-atom distance between the terminal GG residues of WT ISG15 and the USP18 reference contact residue set was 2.8 ± 0.1 Å. In the c.498A>T model, the corresponding distance increased to 36.3 ± 3.2 Å (**Figure 4E**). In line with this possible spatial shift, the GG-related local contacts observed in the WT model were not detected in the c.498A>T model (**Figure 4F**). Root-mean-square deviation (RMSD) and root-mean-square fluctuation (RMSF) analyses indicated that the main changes involved the relative conformation of the complex and dynamic fluctuations of the extended ISG15 C-terminus, rather than broad instability of USP18 itself (**Figures 4G, H**).

We then assessed ISGylation, the ubiquitin-like conjugation of ISG15 to target proteins. Compared with healthy controls, patient PBMCs showed reduction in ISGylation, supporting impaired or altered ISG15 conjugation in primary immune cells (**Figure 3F**). In 293T cells, c.234C>A almost abolished ISGylation, whereas c.498A>T or co-expression of c.234C>A/c.498A>T markedly reduced ISGylation levels (**Figure 4I**). Mechanistically, the C-terminal GG motif of ISG15 serves as the structural basis for both covalent ISGylation and high-affinity non-covalent binding. However, these two functions are biochemically separable-non-covalent binding depends directly on the physical presence and spatial presentation of the GG motif, rather than on ISGylation. Thus, c.498A>T is associated with conformational changes in the ISG15 C-terminus and reduced ISGylation activity. Unlike the truncating allele, the c.498A>T variant does not compromise USP18 protein abundance or the intrinsic binding affinity between ISG15 and USP18. Instead, it exerts its effect primarily by disrupting the spatial presentation of the C-terminal LRLRGG motif, thereby impairing the non-covalent regulatory interface without affecting USP18 stability or complex formation. This variant may therefore contribute to impaired IFN-I negative feedback despite preserved USP18 expression and binding.

### Improvement of IFN-I signaling and clinical manifestations after baricitinib treatment

Given the activation of the IFN-I pathway, the patient was treated with the JAK inhibitor baricitinib. After treatment, CRP, PCT, AST and ALT levels decreased, complement levels recovered, and inflammatory cytokines and chemokines, including IL-8, IL-6, IL-1β and MCP-1, showed a downward trend (**Table 1**; **Figure 5A**). In patient-derived activated T cells, baricitinib pretreatment reduced IFN-α-induced p-STAT1 signaling, as shown by both immunoblotting and flow cytometry (**Figures 5B, C**). Longitudinal transcriptomic analysis showed an overall reduction in IFN-I pathway-related gene expression after treatment. RT-qPCR confirmed decreased expression of IFI44L, OAS1, SIGLEC1 and IFIT1, accompanied by a lower IFN-I score (**Figures 5D–F**). Clinically, the patient’s ulcerative skin lesions improved markedly after treatment (**Figure 5G**).

**Figure 5:**
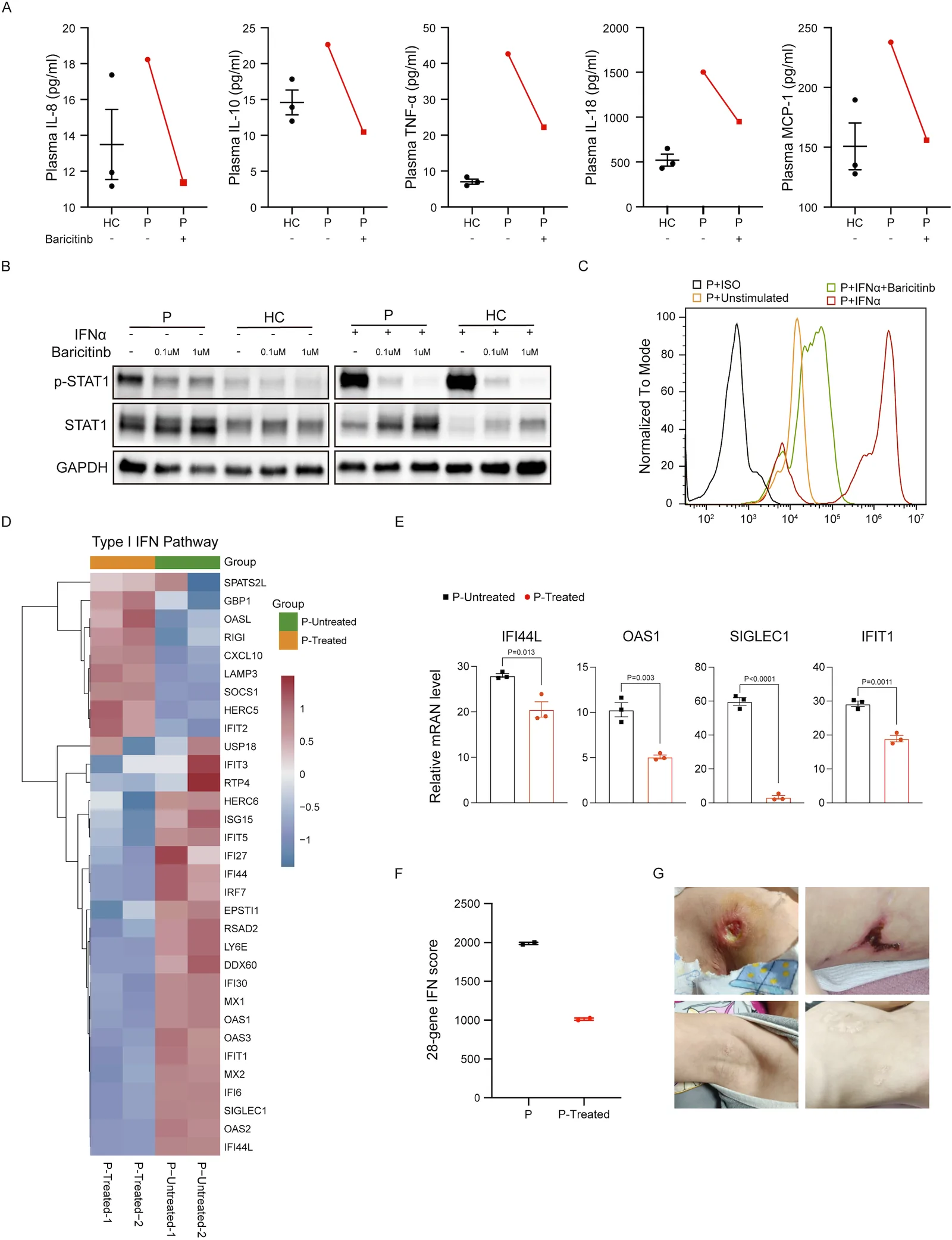
Improvement of IFN-I signaling and clinical manifestations after baricitinib treatment. **(A)** Changes in serum cytokines and chemokines before and after treatment. **(B, C)** Activated T cells from the patient and HC were pretreated with baricitinib and then stimulated with IFN-α, followed by analysis of p-STAT1 levels. **(D)** RNA-seq analysis showing expression of IFN-I pathway-related genes before and after treatment. **(E)** RT-qPCR analysis of ISG expression in PBMCs before and after treatment. **(F)** IFN-I score before and after treatment. **(G)** Representative clinical images showing improvement of skin lesions after treatment. Data are presented as mean ± SEM. Statistical significance was determined using an unpaired two-tailed Student’s t-test. Exact P values are indicated in the graphs.

### Mutation spectrum and clinical features of ISG15 deficiency

We summarized the clinical and genetic data of 30 genetically confirmed patients with ISG15 deficiency (**Table 2**). Cases came from multiple countries and populations. Some occurred in familial clusters or consanguineous families. ISG15 variants were distributed across different protein regions, with variants in the UBL2/C-terminal region being relatively more frequent. Most were nonsense or frameshift variants, consistent with a predominant pattern of protein truncation or loss of function (**Figures 6A, B**). The c.234C>A and c.498A>T variants identified here expand the ISG15 variant spectrum. Notably, the c.498A>T stop-loss variant leads to C-terminal extension and reveals a novel pathogenic mechanism that is distinct from the classical loss-of-function model, suggesting that C-terminal functional abnormalities may represent an independent type of regulatory defect.

**Table 2:** Features of previously and new reported ISG15 deficiency patients.

Case	Country	Variant	Protein	Inheritance	Variant group	BCG vaccination	Skin lesion	Intracranial calcification	Pulmonary manifestations	Ref
P1	Turkey	c.379G>T	p.E127*	Homozygous	UBL2	YES	YES	YES	–	(8)
P2^1^	Iran	c.336_337insG	p.L114fs	Homozygous	UBL2	YES	–	YES	–	(8)
P3^1^	Iran	c.336_337insG	p.L114fs	Homozygous	UBL2	YES	–	YES	–	(8)
P4^2^	China	c.163C>T	p.Q55*	Homozygous	UBL1	–	NO	YES	–	(9)
P5^2^	China	c.163C>T	p.Q55*	Homozygous	UBL1	–	NO	YES	–	(9)
P6^2^	China	c.163C>T	p.Q55*	Homozygous	UBL1	–	NO	YES	–	(9)
P7	US	c.310G>A/c.352C>T	p.V104M/p.Q118*	Compound heterozygous	UBL2	–	YES	YES	NO	(10)
P8^3^	Saudi Arabia	c.4-1G>A	Splicing alterations	Homozygous	Splice-site	YES	YES	YES	–	(10)
P9^3^	Saudi Arabia	c.4-1G>A	Splicing alterations	Homozygous	Splice-site	NO	YES	YES	–	(10)
P10	Spain	c.83T>A/c.284del	p.L28Q/p.T95fs	Compound heterozygous	–	–	YES	YES	–	(10)
P11	Argentina	c.284del/c.297_313del	p.T95fs/p.R99fs	Compound heterozygous	UBL2	YES	YES	YES	YES	(10, 13)
P12^4^	Saudi Arabia	c.4-1G>A	Splicing alterations	Homozygous	Splice-site	–	YES	YES	NO	(14, 21)
P13^4^	Saudi Arabia	c.4-1G>A	Splicing alterations	Homozygous	Splice-site	–	YES	NO	NO	(21)
P14	Saudi Arabia	c.4-1G>A	Splicing alterations	Homozygous	Splice-site	–	NO	YES	YES	(21)
P15^5^	Egypt	c.288C>G	p.Y96*	Homozygous	UBL2	NO	YES	YES	–	(12)
P16^5^	Egypt	c.288C>G	p.Y96*	Homozygous	UBL2	NO	YES	NO	–	(12)
P17^6^	Saudi Arabia	c.463dup	p.R155Pfs	Homozygous	UBL2	YES	YES	YES	–	(22)
P18^6^	Saudi Arabia	c.463dup	p.R155Pfs	Homozygous	UBL2	–	YES	–	–	(22)
P19^6^	Saudi Arabia	c.463dup	p.R155Pfs	Homozygous	UBL2	–	YES	–	–	(22)
P20^6^	Saudi Arabia	c.463dup	p.R155Pfs	Homozygous	UBL2	–	YES	–	–	(22)
P21^7^	China	c.420C>A	p.Y140X	Homozygous	UBL2	NO	NO	YES	YES	(23)
P22^7^	China	c.420C>A	p.Y140X	Homozygous	UBL2	–	YES	NO	NO	(23)
P23^8^	United Kingdom	c.46C>T + 1p36.33del	p.Q16*/1p36.33del	Compound heterozygous	UBL1	–	YES	NO	NO	(24)
P24^8^	United Kingdom	c.46C>T + 1p36.33del	p.Q16*/1p36.33del	Compound heterozygous	UBL1	–	NO	–	–	(24)
P25^9^	Pakistan	ISG15:del/del	–	Homozygous	Whole-gene deletion	YES	YES	YES	YES	(24)
P26^9^	Pakistan	ISG15:del/del	–	Homozygous	Whole-gene deletion	YES	YES	NO	NO	(24)
P27	Brazil	c.83T>A	p.L28Q	Homozygous	UBL1	NO	YES	YES	YES	(25)
P28	Brazil	c.83T>A	p.L28Q	Homozygous	UBL1	NO	YES	NO	–	(25)
P29	China	c.392T>C	p.L131P	Homozygous	UBL2	YES	YES	YES	YES	(26)
P30	China	c.234C>A/c.498A>T	p.C78*/p.*166Y	Compound heterozygous	–	YES	YES	NO	NO	This paper

1,2,3,4,5,6,7,8,9 indicates the same families.

- not mentioned.

*: represents a stop codon.

**Figure 6:**
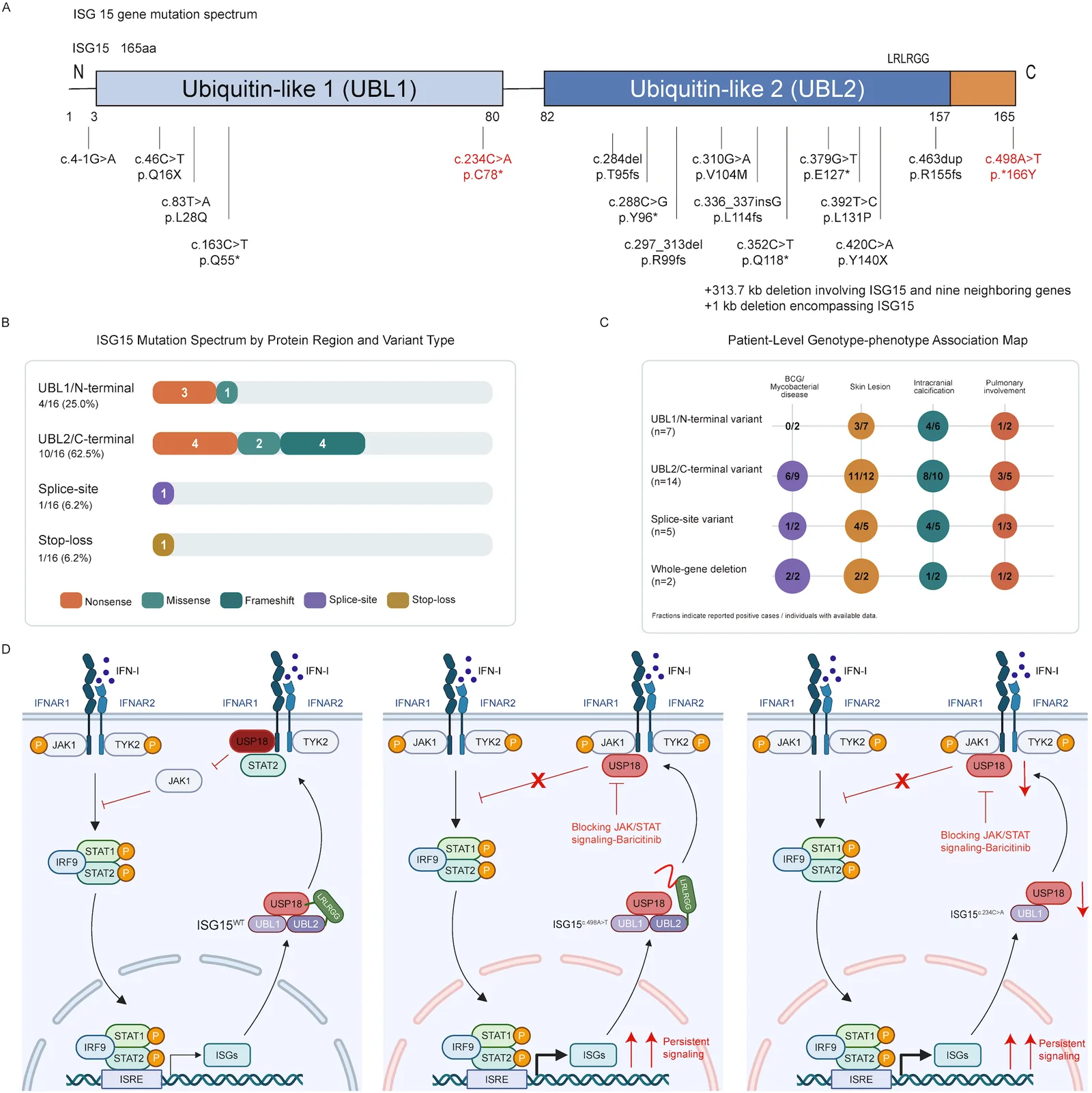
ISG15 variant spectrum and pathogenic mechanisms. **(A)** Schematic representation of ISG15 protein structure and reported ISG15 variants. **(B)** Distribution of reported ISG15 variants by protein region and variant type. **(C)** Patient-level genotype-phenotype association map. **(D)** Proposed mechanism of ISG15 dysfunction caused by patient-derived variants. WT ISG15 stabilizes USP18 and supports negative feedback through proper C-terminal LRLRGG/GG presentation. The c.498A>T stop-loss variant extends the C-terminus, internalizing the terminal GG motif and impairing ISGylation and USP18-mediated feedback, without reducing USP18 levels or disrupting ISG15-USP18 binding *in vitro*. The c.234C>A truncating variant removes the UBL2 domain and LRLRGG/GG motif, leading to USP18 loss and more severe functional impairment. Both variants contribute to persistent IFN-I/JAK-STAT activation and increased ISG expression, which can be partially attenuated by baricitinib.

The major clinical features of ISG15 deficiency included skin lesions, intracranial calcification, pulmonary involvement and BCG/mycobacterial manifestations (**Figure 6C**; **Table 2**). Phenotype frequencies were calculated only from cases in which the corresponding information was explicitly reported in the original publications. Patients carrying compound heterozygous variants spanning different protein regions were not included in the region-based analysis. The currently available cases therefore remain insufficient to establish a stable regional genotype-phenotype correlation.

## Discussion

In this study, we describe a child with ISG15-associated type I interferonopathy caused by compound heterozygous ISG15 variants, including two previously unreported variants. The patient presented with refractory ulcerative skin lesions beginning in infancy, recurrent fever and elevated liver enzymes, without a definite history of mycobacterial disease or intracranial calcification, but with persistent activation of the IFN-I pathway. To investigate the functional consequences of these variants on the ISG15-USP18 regulatory axis, we examined USP18 expression in patient PBMCs and found it to be decreased compared with healthy controls, supporting impaired ISG15-mediated negative feedback.

The two ISG15 alleles identified in this patient appeared to have different functional consequences. To place these alleles within the known spectrum of ISG15 deficiency, we reviewed reported variants and found disease-associated changes in the UBL1/N-terminal region, UBL2/C-terminal region, splice sites, and stop-codon region, with a relative enrichment in the UBL2/C-terminal region. Nonsense and frameshift variants predominated, consistent with an enrichment of protein-truncating or otherwise severely damaging alleles (10, 12). In this context, the c.234C>A variant supports a classic loss-of-function mechanism, as it is predicted to remove UBL2 and the C-terminal LRLRGG motif and was associated with markedly impaired ISGylation in functional assays and USP18-related ISG15 biology (9).

The c.498A>T variant is different: it is a stop-loss mutation that hasn’t been reported before and seems to work through another mechanism. It doesn’t simply delete the LRLRGG sequence. Instead, losing the stop codon is predicted to add extra amino acids to the C-terminus, converting the normally terminal GG residues into internal residues. Because ISGylation requires the LRLRGG motif to be exposed at the mature ISG15 C-terminus for recognition, activation and conjugation by the E1/E2/E3 enzyme cascade, this internalization provides a direct explanation for the reduced ISGylation observed in our research. Importantly, c.498A>T alone did not decrease USP18 expression in 293T cells and retained a detectable interaction with USP18, whereas patient PBMCs showed reduced USP18 expression in the compound heterozygous context. These findings suggest that the patient phenotype likely reflects both reduced USP18 related to the truncating allele and impaired C-terminal functional presentation caused by the stop-loss allele.

Recent cell-based studies on ISG15 C-terminal function support this interpretation (17). Engineered mutants lacking the C-terminal GG residues or carrying GG-to-AA substitutions can still stabilize USP18 to some extent, but fail to fully restore USP18-mediated suppression of IFN-I signaling. These findings indicate that USP18 protein stability alone is insufficient to ensure intact negative feedback regulation. Structural studies show that USP18 recognizes ISG15 in two steps (18). First, ISG15’s Trp123 binds to the hydrophobic pocket of USP18 IBB-1. Then the USP18 switching loop rearranges, placing the C-terminal LRLRGG tail near USP18’s active-site region. The c.498A>T variant described here differs from these engineered mutants: it retains the LRLRGG sequence but is predicted to alter its terminal positioning and spatial presentation. Thus, the presence of the LRLRGG sequence alone may be insufficient. The motif also needs to be correctly positioned at the mature C-terminus for ISG15 to support USP18-mediated IFN-I signaling and ISGylation. Combined with data from engineered C-terminal mutants, our findings support the idea that an abnormal ISG15 C-terminus can impair IFN-I regulation in a real patient setting. However, because our structural results are predictive, this mechanism should be considered a plausible model that requires further validation by structural or biochemical approaches. Although impaired ISG15-USP18 negative feedback and reduced ISGylation appear central in this case, additional ISGylation-dependent immune pathways may also contribute to disease pathogenesis. Limited patient tissue precluded ISGylome-wide, proteomic analyses, or subcellular localization analyses to identify specific affected targets.

Clinically, our review of 30 genetically confirmed patients with ISG15 deficiency showed marked clinical heterogeneity. Reported manifestations included BCG/mycobacterial disease, skin lesions, intracranial calcification and pulmonary involvement. The patient in this study had no definite mycobacterial disease or intracranial calcification, but instead presented mainly with early-onset, treatment-refractory ulcerative skin disease and an elevated IFN-I signature. Thus, ISG15-associated interferonopathy should be considered in children with early-onset, recurrent or treatment-refractory ulcerative skin lesions, even in the absence of typical infection history or neuroimaging abnormalities. This case also highlights the skin as a potentially important target tissue of dysregulated ISG15-IFN-I signaling, in line with recent reports of severe inflammatory skin disease caused by ISG15 variants.

Given the persistent IFN-I activation and skin inflammation in this patient, we initiated treatment with the JAK inhibitor baricitinib, which led to reduced IFN-I-related molecular markers and marked improvement of ulcerative skin lesions and liver enzyme abnormalities. Together with previous reports of JAK inhibitor use in patients with ISG15 deficiency, our findings further support the JAK-STAT pathway as a therapeutic target in ISG15-associated interferonopathy (19, 20). Current management therefore remains individualized, including infection prevention and treatment, anti-inflammatory care, management of skin lesions, and targeted inhibition of excessive IFN-I/JAK-STAT signaling when clinically appropriate.

This study has several limitations. First, limited patient sample availability precluded comprehensive analyses of ISGylation targets and subcellular localization in disease-relevant tissues. Second, the structural findings were based on computational modeling and therefore require experimental validation. Third, parental RNA-seq data suggest possible gene-dosage effects or subclinical molecular changes, but cannot establish clinical disease in heterozygous carriers without protein-level of functional evidence.

In summary, this study reports a child with compound heterozygous ISG15 variants and identifies c.498A>T as a previously unreported stop-loss variant in ISG15. This work provides patient-level evidence supporting a model in which abnormal functional presentation of the ISG15 C-terminus may contribute to impaired IFN-I negative feedback and ISGylation dysfunction. These findings complement the classical model of ISG15 loss leading to USP18 instability by suggesting that C-terminal spatial presentation may also influence ISG15-dependent IFN-I regulation. Potential functional biomarkers for ISG15-associated disease include the IFN-I signature, the 28-gene IFN score, and p-STAT1 signaling. This study expands the genetic and mechanistic spectrum of ISG15 deficiency and provides further rationale for considering ISG15 genetic testing and JAK-STAT-targeted therapy in children with early-onset refractory ulcerative skin disease accompanied by IFN-I pathway activation.

## Data Availability

The transcriptome data reported in this study have been submitted to the CNSA (CNGB Sequence Archive) at CNGBdb under accession number CNP0009635. De-identified data are made available through a controlled access process. Other data that support the study’s conclusions can be obtained from the corresponding author upon reasonable request.
