# Supplementary material for: A novel stop-loss variant in ISG15 causes C-terminal dysfunction and impairs USP18-mediated negative feedback
Source: Front Immunol. 2026 Sep 17;17:1916670. doi: 10.3389/fimmu.2026.1916670 (PMC13627440; doi:10.3389/fimmu.2026.1916670)
Supplement: Supplementary file 1 [file Supplementaryfile1.docx]

Supplemental Material

A novel stop-loss variant in ISG15 causes C-terminal dysfunction and impairs USP18-mediated negative feedback

Wanyu Zhang^1,†^, Xiongbin Chen^1,†^, Yulu Li^1^, Xingfeng Yao^2^, Jinyan Huang^1^, Yijiu Gao^1^, Wenlu Xing^1^, Dan Lu^1^, Mengyue Deng^1^, Zhou Shu^1^, Huawei Mao^1,3,4,*^

^1^Department of Rheumatology & Immunology, Beijing Children's Hospital of Capital Medical University, National Center for Children's Health of China, Beijing, China

^2^Department of Pathology, Beijing Children’s Hospital, Capital Medical University, National Center for Children’s Health (NCCH), Beijing, 100045, China

^3^Ministry of Education Key Laboratory of Major Diseases in Children, Beijing Key Laboratory for Genetics of Birth Defects, Beijing, China

^4^Beijing Key Laboratory of Precision Diagnosis and Treatment of Pediatric Immune Diseases, Beijing, China

†These authors contributed equally to this work.

*Correspondence:

Huawei Mao

Department of Rheumatology & Immunology, Beijing Children's Hospital of Capital

Medical University, National Center for Children's Health of China

No. 56 Nanlishi Road, Xicheng District, Beijing 100045, China

Tel: +86 13928459556

Email: maohwei@qq.com

Conflict of interest statement: The authors have declared that no conflict of interest exists.

Supplementary tables

Table S1 Peripheral lymphocyte subsets of the patient.

| Subset | Percentage | Reference range | Absolute counts(/ul) | Reference range |
| --- | --- | --- | --- | --- |
| T cells | 76.9 | 53.88–72.87 | 2572 | 1794–4247 |
| CD4^+^T | 35.4 | 24.08–42.52 | 1184 | 902–2253 |
| Naive CD4^+^T | 53 | 46.14–84.40 | 628 | 472–1760 |
| Temra CD4^+^T | 0 | 0.00–1.36 | 0 | 0–22 |
| CM CD4^+^T | 40.7 | 13.88–48.12 | 482 | 212–735 |
| EM CD4^+^T | 6.3 | 0.94–6.46 | 75 | 15–87 |
| CD8^+^T | 35 | 19.00–32.51 | 1171 | 580–1735 |
| Naive CD8^+^T | 87.6 | 36.80–83.16 | 1026 | 356–1095 |
| Temra CD8^+^T | 3.2 | 0.84–33.02 | 37 | 9–440 |
| CM CD8^+^T | 8.2 | 5.18–31.66 | 96 | 56–406 |
| EM CD8^+^T | 1.1 | 0.70–11.22 | 13 | 6–145 |
| γδT | 9.1 | 4.94–17.98 | 212 | 114–539 |
| TCRαβ+DNT | 1.1 | 0.37–1.80 | 26 | 9–57 |
| B cells | 14.5 | 13.23–26.39 | 522 | 461–1456 |
| Memory B | 28.6 | 2.98–14.18 | 149 | 26–124 |
| Naive B | 35.2 | 65.54–86.62 | 184 | 323–1080 |
| Transitional B | 4.4 | 5.24–17.22 | 23 | 35–172 |
| Plasmablast B | 1.1 | 0.5–7.06 | 6 | 4–63 |
| NK cells | 9.1 | 7.21–20.90 | 327 | 270–1053 |
| DNT | 1.1 | 0.37-1.80 | 26 | 9-57 |
| CD4:CD8 | 1.01 | 0.90–2.13 |  |  |

Table S2 Analysis of helper T cell subsets in the patient

| Subset | Percentage (%) | Reference range (%) |
| --- | --- | --- |
| CD3^+^T | 70.76 | 50.00–84.00 |
| CD3^+^CD4^+^ T | 43.83 | 30.00–67.00 |
| CD3^+^CD8^+^ T | 50.49 | 23.00–50.00 |
| CD3^+^CD8^-^ T | 49.51 | 50.00–75.00 |
| CD3^+^CD8^-^IFN-γ+ T cells (Th1-like) | 10.74 | 6.50–28.00 |
| CD3^+^CD4^+^CD25^+^FOXP3^+^T (Treg) | 8.47 | 4.10–9.40 |
| CD3^+^CD8^-^IL-4^+^ T(Th2-like) | 0.14 | 0.30–2.20 |
| CD3^+^CD8^-^IL-17A^+^ T cells (Th17-like) | 1.11 | 0.20–2.40 |

Table S3 Summary of primers used in this study.

| Primer | Sequence |
| --- | --- |
| *IFI27*-F | TTGTGGCTACTCTGCAGTCA |
| *IFI27*-R | CAATGGCAGACCCAATGGAG |
| *IFI44L*-F | GCCACCGTCAGTATTTGGAA |
| *IFI44L*-R | GGACCCACCAAAAGAATACGAA |
| *IFI44*-F | AGTTGGTAAACGCTGGTGTG |
| *IFI44*-R | ACCTCACAGGCTCACATCTC |
| *MX1-F* | TCCAGCCACATCCCTTTGAT |
| *MX1-R* | TCCTTCAGGAACTTCCGCTT |
| *OAS1-F* | TTCCGCATGCAAATCAACCA |
| *OAS1-R* | GACACACACACAGGGTAGGA |
| *IFIT1*-F | TGCTTGAAGTGGACCCTGAA |
| *IFIT1*-R | TGGATTTAAGCGGACAGCCT |
| *RSAD2*-F | GGGAGAGGTGGTTCCAGAAT |
| *RSAD2*-R | CCACGGCCAATAAGGACATT |
| *SIGLEC1*-F | CTTCTGTGAGGTGCAGAACG |
| *SIGLEC1*-R | CACAGAGCAGTGAAGGATGC |
| *GAPDH*-F | ACAACTTTGGTATCGTGGAAGG |
| *GAPDH*-R | GCCATCACGCCACAGTTTC |

Abbreviations: F, forward; R, reverse; qPCR, quantitative reverse transcription-PCR.

Table S4 Summary of antibodies used in this study.

| **Antibodies** | **Source** | **Identifier** |
| --- | --- | --- |
| DYKDDDDK-Tag (3B9) Mouse mAb | Abmart | M20008 |
| GAPDH (14C10) Rabbit mAb | CST | 2118 |
| ISG15 Polyclonal antibody (full-length) | Proteintech | 15981-1-AP |
| ISG15 Rabbit mAb (C-terminal antibody) | CST | 46155 |
| USP18 (D4E7) Rabbit mAb | CST | 4813 |
| Phospho-Stat1 (Tyr701) (58D6) Rabbit mAb | CST | 9167 |
| Stat1 (D1K9Y) Rabbit mAb | CST | 14994 |

Abbreviations: mAb, monoclonal antibody; CST, Cell Signaling Technology.
