# Supplementary material for: A novel stop-loss variant in ISG15 causes C-terminal dysfunction and impairs USP18-mediated negative feedback
Source: Front Immunol. 2026 Sep 17;17:1916670. doi: 10.3389/fimmu.2026.1916670 (PMC13627440; doi:10.3389/fimmu.2026.1916670)
Supplement: Supplementary file 2 [file Supplementaryfile2.docx]

**Supplementary Figure 1. Antibody validation and full-length blots.**

(A) Full-length Western blots of 293T cells overexpressing N-terminally FLAG-tagged EV, WT ISG15, c.234C>A truncation, c.498A>T extension, or the c.234C>A/c.498A>T combination. Blots were probed with full-length anti-ISG15 and anti-Flag.

(B) The same overexpression lysates were probed with C-terminal anti-ISG15 alongside anti-Flag. The C-terminal antibody detected WT and extended ISG15, but not the truncated form, whereas anti-Flag detected all constructs.

(C) Endogenous ISG15 in patient and control PBMCs after IFN-α stimulation (0, 12, 24, 48 h), detected with C-terminal anti-ISG15.
